# Expression of the Long Noncoding RNA GAS5 Correlates with Liver Fibrosis in Patients with Nonalcoholic Fatty Liver Disease

**DOI:** 10.3390/genes11050545

**Published:** 2020-05-13

**Authors:** Man-Hoon Han, Jee Hyun Lee, Gyeonghwa Kim, Eunhye Lee, Yu Rim Lee, Se Young Jang, Hye Won Lee, Jae Min Chun, Young Seok Han, Jun Sik Yoon, Min Kyu Kang, Won Kee Lee, Young Oh Kweon, Won Young Tak, Soo Young Park, Jung Gil Park, Keun Hur

**Affiliations:** 1 Department of Pathology, School of Medicine, Kyungpook National University, Kyungpook National University Hospital, Daegu 41944, Korea; one-many@hanmail.net; 2 Department of Biochemistry and Cell Biology, Cell and Matrix Research Institute, School of Medicine, Kyungpook National University, Daegu 41944, Korea; jihyunlee.knu@gmail.com (J.H.L.); aoet111@gmail.com (G.K.); eunhye7832@naver.com (E.L.); 3 Department of Internal Medicine, School of Medicine, Kyungpook National University, Kyungpook National University Hospital, Daegu 41944, Korea; deblue00@naver.com (Y.R.L.); magnolia1103@naver.com (S.Y.J.); yokweon@knu.ac.kr (Y.O.K.); wytak@knu.ac.kr (W.Y.T.); psyoung0419@gmail.com (S.Y.P.); 4 Department of Pathology, Dongsan Medical Center, School of Medicine, Keimyung University, Daegu 42601, Korea; hoongirl82@naver.com; 5 Department of Surgery, School of Medicine, Kyungpook National University, Kyungpook National University Hospital, Daegu 41944, Korea; mong0101@dreamwiz.com (J.M.C.); gshyskhk@hanmail.net (Y.S.H.); 6 Department of Internal Medicine, Busan Paik Hospital, Inje University College of Medicine, Busan 74392, Korea; yojusi@naver.com; 7 Department of Internal Medicine, College of Medicine, Yeungnam University, Daegu 42415, Korea; kmggood111@naver.com; 8 Medical Research Collaboration Center in KNUH and School of Medicine, Kyungpook National University, Daegu 42415, Korea; wonlee@knu.ac.kr

**Keywords:** noncoding RNA, biomarker, lncRNA GAS5, nonalcoholic fatty liver disease, liver fibrosis

## Abstract

Background: Advanced liver fibrosis is the most important prognostic factor in nonalcoholic fatty liver disease (NAFLD). The long noncoding RNA (lncRNA), growth arrest-specific transcript 5 (GAS5), is associated with the inhibition of liver fibrogenesis, and its levels are decreased in cirrhotic liver. Methods: We analyzed 51 patients with NAFLD, the diagnosis of which was confirmed by liver biopsy. Expression of GAS5 in both the liver and plasma of the patients was analyzed using a quantitative real-time polymerase chain reaction according to the fibrosis stage. Results: Plasma GAS5 expression was significantly higher in patients with advanced fibrosis than in those without. As the fibrosis progressed, GAS5 expression in plasma increased, with the exception of that in cirrhotic livers. Plasma levels of GAS5 were lower in patients with cirrhosis than in those with advanced fibrosis. Conclusion: Elevated circulating levels of the lncRNA GAS5 are associated with the progression of liver fibrosis prior to the development of cirrhosis.

## 1. Introduction

Nonalcoholic fatty liver disease (NAFLD) is the most common chronic liver disease, affecting 25% of individuals in Western countries [1]. The rise in the number of obese people over the past three decades has led to an increase in the incidence of NAFLD, associated with elevated healthcare costs [2]. Inflammation, which can progress to cirrhosis, has been observed in 20–25% of NAFLD patients. The pathology of NAFLD ranges widely, from simple steatosis without liver injury to nonalcoholic steatohepatitis (NASH) with or without advanced fibrosis and cirrhosis, which can cause liver cancer [3]. Due to its variable natural history, it is difficult to identify the risk factors associated with poor prognosis among patients with NAFLD. Recent studies have revealed that the presence of advanced fibrosis is independently associated with overall mortality, regardless of NASH or a high NAFLD activity score (NAS) [4,5]. Although invasive, liver biopsy is the only confirmatory procedure by which NAFLD grade and stage can be ascertained. Using demographic characteristics and biochemical parameters, several studies have proposed noninvasive methods of assessing fibrosis in patients with NAFLD. However, given that such variables are indirect markers of fibrosis, their diagnostic accuracy is insufficient for clinical applications [6]. Thus, the identification of a direct marker of fibrosis in patients with NAFLD measurable via a noninvasive test is important.

Long noncoding RNAs (lncRNAs) are defined as RNA molecules longer than 200 nucleotides that are not translated into proteins [7]. They are known to play a role in the regulation of gene expression by diverse mechanisms, such as chromatin modification, transcription, and post-transcriptional processing [8]. Growth arrest-specific transcript 5 (GAS5), which was originally isolated from mouse NIH 3T3 cells by subtractive hybridization, plays various tumor-suppressive roles in several cancers, including those of the breast, stomach, prostate, and lung [9,10,11,12,13]. Several recent studies have reported that the plasma levels of GAS5 are associated with diabetes and coronary artery disease [14,15]. In addition, a mechanism by which GAS5 inhibits liver fibrogenesis by acting as a competing endogenous RNA has recently been demonstrated [16].

In the present study, we evaluated GAS5 expression in both the tissue and plasma of patients with NAFLD using a real-time polymerase chain reaction (PCR). In addition, to evaluate its changes during the progression of fibrosis, GAS5 expression was analyzed according to the liver fibrosis stage.

## 2. Materials and Methods

### 2.1. Patient Samples

A total of 51 tissue and plasma samples were collected from patients with NAFLD at a tertiary hospital between January 2014 and June 2016. All tissue samples were collected using ultrasound-guided percutaneous liver biopsy on the same day that plasma samples were collected to avoid any confounding factors. Patient biochemical parameters were also measured on this day. Written informed consent was obtained from all patients, and this study was approved by the Ethical Committees of our center. The institutional review board (IRB) number of our study is KNUH-2018-05-025-003.

### 2.2. Diagnosis of NAFLD and Pathologic Evaluations

NAFLD was defined as the accumulation of at least 5% hepatic fat, as determined by histology, without significant alcohol consumption. Other possible causes for chronic hepatitis were excluded by tests of a serologic marker of viral hepatitis and examination of medical records. A single well-trained pathologist reviewed all specimens to avoid inter-observer variation. NASH was diagnosed according to current guidelines [17]. The NAFLD activity score (NAS) and fibrosis stage were evaluated according to the system devised by the Pathology Committee of the NASH Clinical Research Network [18]. An NAS above 4 was defined as severe NAFLD.

### 2.3. RNA Extraction

Total RNA was extracted from patient tissue samples using QIAzol Lysis Reagent (Qiagen, Hilden, Germany). To collect plasma samples, peripheral blood was separated by centrifugation at 3000 rpm for 10 min at 4 °C. Total RNA was then extracted from plasma using a miRNeasy Serum/Plasma Kit (Qiagen), as described previously [19]. The quality and quantity of the extracted total RNA were measured with a NanoDrop ND-1000 spectrophotometer (NanoDrop Technologies, Wilmington, DE, USA).

### 2.4. Quantitative Real-Time PCR

For tissue samples, 800 µg of the total RNA was used for reverse transcription with a High-Capacity cDNA Reverse Transcription Kit (Thermo Fisher Scientific Inc., Waltham, MA, USA) according to the manufacturer’s protocol. The resulting cDNA was diluted with 80 µL of distilled water, and 1 µL of this solution was used in the experiment. For plasma samples, 500 µg of the total RNA was used for reverse transcription, the product of which was also diluted with 80 µL of distilled water. Four microliters of this diluted cDNA was then used. Quantitative real-time PCR was performed using SYBR Green PCR Master Mix (Thermo Fisher Scientific Inc.), and each sample was processed in triplicate. The PCR conditions were as follows: 2 min at 50 °C and 10 min at 95 °C, followed by 45 cycles of 15 s at 95 °C and 60 s at 60 °C. The *GAPDH* gene was used as an endogenous control, and the relative expression of GAS5 was determined using the 2^−ΔΔCt^ method. The following primers were used for the detection of each gene: sense- lncRNA-GAS5 (5′-TTGAAAGGGTCTTGCCTCAC-3′) and antisense-lncRNA- GAS5 (5′-GGATCACTTGAGCCCAGAAG-3′) (Figure S1); and sense- GAPDH (5′-GGAAGGTGAAGGTCGGAGTC-3′) and antisense-GAPDH (5′-GTTGAGGTCAATGAA GGGGTC-3′).

### 2.5. Statistical Analysis

All data are expressed as means ± standard deviations or counts with percentages. Statistically significant differences between patients were determined using the chi-square test, Student’s *t*-test, and Fisher’s exact test. Differences in the expression of the target lncRNA were identified using the 2^−ΔΔCt^ method. The correlation between GAS5 and fibrosis was analyzed using Spearman’s correlation coefficient. *P*-values less than 0.05 were considered statistically significant. All statistical analyses were performed using R version 3.2.2 (R Foundation for Statistical Computing, Vienna, Austria).

## 3. Results

### 3.1. Tissue and Plasma GAS5 Was UpRegulated in Patients with Advanced Liver Fibrosis

**Figure 1:**
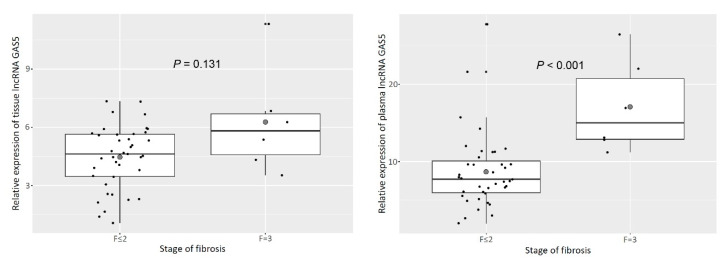
Tissue and plasma lncRNA GAS5 was upregulated in patients with nonalcoholic fatty liver disease and advanced fibrosis compared to those without advanced fibrosis.

The characteristics of the patients, separated according to the presence or absence of significant fibrosis, are shown in Table 1. The patients with advanced fibrosis were significantly older, had lower platelet counts and higher aspartate aminotransferase (AST) levels, and were more likely to have diabetes than those without advanced fibrosis. However, the other characteristics tested did not significantly differ between these groups and thus were not associated with fibrosis stage. In terms of GAS5 expression analyses, plasma GAS5 was significantly elevated in patients with advanced fibrosis compared to those without (F ≤ 2: 7.7 (6.0–10.1); F = 3: 15.0 (12.8–22.0); *P* < 0.001, Figure 1). Unexpectedly, tissue GAS5 expression did not show any significant difference in advanced fibrosis patients (F ≤ 2: 4.6 (3.5–5.6); F = 3: 5.8 (4.3–6.8); *P* = 0.131). In addition, expression of GAS5 did not significantly differ according to the degree of steatosis or inflammation or the presence or severity of NASH in either tissue or plasma (Table 2).

### 3.2. Tissue and Plasma GAS5 Increased as Fibrosis Progressed in Patients with NAFLD

**Figure 2:**
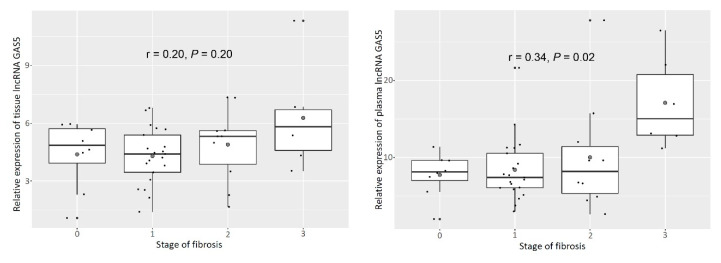
Relative expression of tissue and plasma lncRNA GAS5 according to stage of liver fibrosis. As a positive correlation was observed between the age of the patients and the stage of fibrosis, expression of tissue and plasma GAS5 was also positively correlated with age (age: r = 0.40, *P* = 0.01; tissue: r = 0.41, *P* < 0.01; plasma: r = 0.32, *P* = 0.03, Figure 3).

To evaluate the relationship between GAS5 and fibrosis stage, we analyzed its expression during each stage of fibrosis, with the exception of cirrhosis. Prior to the development of cirrhosis, GAS5 tissue expression positively correlated with fibrosis stage (r = 0.20, *P* = 0.20, Figure 2). In addition, a clearer positive correlation was observed between plasma levels and fibrosis stage (r = 0.34, *P* = 0.02, Figure 2).

### 3.3. Plasma GAS5 Was Downregulated in Patients with Cirrhosis

**Figure 4:**
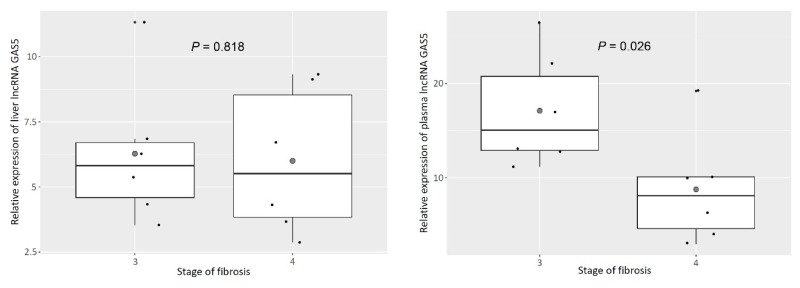
Relative expression of tissue and plasma lncRNA GAS5 between advanced fibrosis and cirrhosis.

Although GAS5 was upregulated in tissue and plasma as liver fibrosis progressed, its expression in plasma was significantly downregulated in patients with NAFLD who had developed cirrhosis (F = 3: 15.0 (12.8–22.0); F = 8.1 (4.0–10.1); *P* = 0.026, Figure 4). However, the tissue levels of GAS5 did not significantly differ between patients with advanced fibrosis and those with cirrhosis (F = 3: 5.8 (4.3–6.8); F = 4: 5.5 (3.7–9.1); *P* = 0.818, Figure 4).

## 4. Discussion

**Figure 5:**
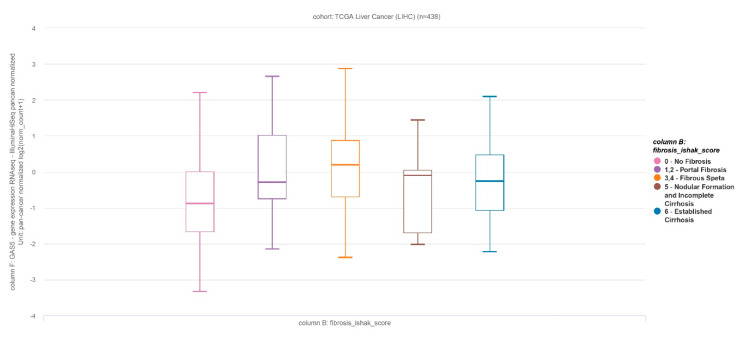
Relative expression of lncRNA GAS5 in The Cancer Genome Atlas (TCGA) liver cancer cohort (one-way ANOVA, *P* = 0.001877).

It has been suggested that the lncRNA GAS5 suppresses hepatocellular carcinoma cell migration and invasion as well as liver fibrosis [20,21,22]. The roles of GAS5 in benign liver disease have been reported in hepatitis C virus (HCV) replication and liver fibrosis. GAS5 binds the NS3 protein, which is a multifunctional protein with serine protease activity to inhibit HCV replication [23]. A previous report revealed that GAS5 is downregulated in liver cirrhosis and forms a reciprocal repressive regulatory loop with miR-222. miR-222 exerts a suppressive effect on p27, a protein that acts to restrict the proliferation of hepatic stellate cells [16]. In addition, another recent study reported that lncRNA GAS5 plays the role of sponge for miR-23a to repress hepatic fibrosis via the PI3K/Akt/mTOR pathway and upregulation of Snail in the CCl4-induced rat model [24]. However, contrary to previous observations, we found that the expression of GAS5 positively correlated with the progression of liver fibrosis. This relationship was evident with respect to both liver tissue and plasma GAS5 levels and was more apparent with regard to the latter. However, as liver fibrosis progressed towards cirrhosis, plasma GAS5 was seen to be abruptly downregulated in the present study, similar to the results of the previous investigation. Cirrhosis represents the final stage of liver disease, which is characterized by the distortion of the hepatic architecture and the loss of liver function. Interestingly, we also observed elevated GAS5 expression in fibrosis rather than non-fibrosis, whereas GAS5 expression decreased in cirrhosis, as established in The Cancer Genome Atlas (TCGA) public database analysis (Figure 5). Thus, it can be hypothesized that plasma GAS5 might be increased to compensate advanced fibrosis for regression of liver fibrosis. We believe that the suppressive effect of GAS5 on liver fibrosis may be maintained until the development of cirrhosis.

The two main mechanisms of lncRNA have been suggested to involve regulating liver fibrosis by acting as a competing endogenous RNA (ceRNA) and direct binding protein [25]. Like GAS5, lncRNA, including lncRNA-p21, PVT1, HOTAIR, and MALAT1, acts as a ceRNA regarding liver fibrosis [26]. lncRNA-p21 inhibits the activation of hepatic stellate cells (HSCs), sponging miR-17-5p to inhibit WIF1 through the Wnt/β-catenin pathway or the miR-181b-PTEN signaling cascade [27,28]. The PVT1 binds competitively with miR-152 to repress the activation of HSCs through enhancing PTCH1 methylation, which is a negative regulator of the Hedgehog pathway [29]. HOTAIR acts as a ceRNA to sponge miR-29b, which induces PTEN methylation via the attenuation of DNMT2b contributing to liver fibrosis [30]. MALAT1 also acts as a ceRNA to sponge miR-101b, which mediates Rac1 expression contributing to liver fibrosis [31]. All of these lncRNAs mediate hepatic fibrogenesis through sponging microRNA, which acts as a ceRNA. Unlike GAS5, other lncRNAs, including MEG3, SCARNA10, linc-SCRG1, lnc-LFAR1, and H19 targeting SMO (MEG3), PRC2 (SCARNA10), Tristetraprolin (linc-SCRG1), Smad2/3(lnc-LFAR1), and MeCP2, ERK1/2, ZEB1, and let7, bind protein (H19) mediate hepatic fibrogenesis with direct binding protein [25]. Recently, in an NAFLD rat model using HULC small interfering RNA, downregulated lncRNA HULC ameliorated liver fibrosis and hepatocyte apoptosis by inhibiting the MAPK signaling pathway [32]. Though the profile of miRNA was not evaluated in this study, HULC is known to modulate abnormal lipid metabolism in hepatoma cells through an miR9-medicated RXRA signaling pathway [33]. Therefore, HULC can be a promising therapeutic target of an NAFLD-associated HCC like GAS5.

GAS5 is also known to inhibit the glucocorticoid receptor by acting as a decoy glucocorticoid receptor element. Moreover, it has been reported that its tissue levels are altered in various autoimmune, inflammatory, and infectious diseases, including systemic lupus erythematosus (SLE), rheumatoid arthritis, multiple sclerosis, sarcoidosis, inflammatory bowel disease, bacterial sepsis, and human immunodeficiency virus type 1 and influenza virus infections [34,35]. GAS5 is also positively regulated by proinflammatory mediators in the respiratory system [36]. However, in our study, GAS5 expression in tissue and plasma did not significantly differ according to the degree of inflammation observed. We conjecture that altered GAS5 levels may not affect inflammation in NASH because this disease is not associated with the glucocorticoid response.

The role of circulating GAS5 has been reported in several studies. The level of this lncRNA in circulation has been identified as a potential biomarker for the prediction of survival in glioblastoma, treatment response in breast, head, and neck cancers, and the diagnosis of non-small-cell lung cancer [13,37,38,39]. mTOR negatively regulates GAS5 expression, which induces tumor cell proliferation. Thus, a decreased level of circulating GAS5 may be an indicator of better survival or treatment response in cancer. However, only limited information is available concerning circulating GAS5 in other contexts. One study has reported that the absolute level of serum GAS5 is a good indicator of diabetes, with a value below 10 ng/μL supporting the diagnosis of this disease [14]. Moreover, combinations of GAS5 and linc0597 or lnc-DC have been identified as possible diagnostic markers of SLE [40]. We believe that our results indicate a possible noninvasive marker of liver fibrosis in patients with NAFLD.

Our study had certain limitations. First, the liver cirrhosis etiology in our investigation was quite different from that of previous functional studies. Most of the human cirrhotic tissues examined in prior work have been associated with hepatitis B virus infection, which may be a confounding factor. Second, the number of patients with advanced fibrosis and cirrhosis was relatively small. However, plasma GAS5 levels were positively correlated as the stage of fibrosis increased, supporting the validity of our results. This was the first study to reveal that levels of GAS5 differ during the progression of fibrosis and development of cirrhosis. The mechanism underlying these effects could not be determined in the present work; thus, it awaits further evaluation in future investigations.

In conclusion, we found that as liver fibrosis progressed, plasma and tissue GAS5 levels increased, whereas the development of cirrhosis was associated with the downregulation of plasma GAS5 in patients with NAFLD.

## Figures and Tables

**Figure 3:**
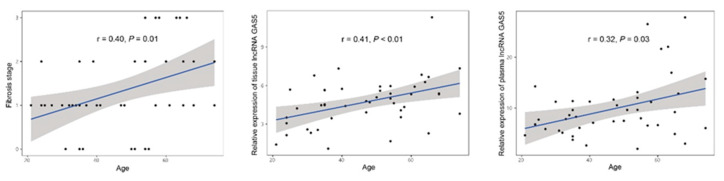
Stage of liver fibrosis and relative expression of tissue and plasma lncRNA GAS5 according to age of the patients.

**Table 1:** Baseline characteristics of the patients.

Characteristic	F ≤ 2 (*N* = 39)	F = 3 (*N* = 6)	*P*-Value
Male (%)	23 (59.0)	2 (33.3)	0.462
Age, year	41.0 (33.5–54.5)	60.5 (57.0–64.0)	0.015 *
Steatosis			0.284
5–33% (%)	13 (33.3)	4 (66.7)	
>33–66% (%)	16 (41.0)	1 (16.7)	
>66% (%)	10 (25.6)	1 (16.7)	
Lobular inflammation			0.496
<2 foci per 200 × field (%)	25 (64.1)	4 (66.7)	
2–4 foci per 200 × field (%)	12 (30.8)	1 (16.7)	
>4 foci per 200 × field (%)	2 (5.1)	1 (16.7)	
Ballooning			0.144
None (%)	8 (20.5)	0 (0.0)	
Few ballooned cells (%)	20 (51.3)	2 (33.3)	
Many cells/prominent ballooning (%)	11 (28.2)	4 (66.7)	
Nonalcoholic steatohepatitis	31 (79.5)	6 (100.0)	0.516
NAS ≥ 5 (%)	17 (43.6)	4 (66.7)	0.538
Weight, kg	79.0 (70.0–97.0)	72.0 (59.3–84.0)	0.226
BMI, kg/m^2^	27.8 (26.1–32.4)	27.9 (26.5–30.4)	0.694
Systolic blood pressure, mmHg	138 (127–148)	132.0 (132–133)	0.573
Hypertension	19 (48.7)	4 (66.7)	0.704
Diabetes	10 (25.6)	5 (83.3)	0.020 *
Platelets, ×10^3^/mm	240.0 (200.0–286.0)	167.0 (142.0–192.0)	0.023
AST, IU/L	63.0 (51.5–82.0)	119.0 (74.0–165.0)	0.019 *
ALT, IU/L	96.0 (70.5–128.5)	76.0 (58.0–173.0)	0.593
Bilirubin, mg/dL	0.6 (0.5–0.7)	0.7 (0.7–0.9)	0.681
Albumin, g/dL	4.6 (4.4–4.8)	4.5 (4.1–4.7)	0.350
Gamma GTP, mg/dL	60.0 (46.0–123.0)	61.0 (52.0-92.0)	0.820
Creatinine, mg/dL	0.8 (0.7–0.9)	0.7 (0.7–0.9)	0.494
Fasting blood glucose, mg/dL	108.0 (98.0–123.0)	118.5 (113.0–127.0)	0.142
Total cholesterol, mg/dL	183.0 (164.0–204.0)	167.5 (160.0–211.0)	0.687
HDL, mg/dL	38.5 (31.0–49.0)	48.5 (43.0–56.0)	0.098
LDL, mg/dL	118.5 (93.0–145.0)	108.5 (100.0–151.0)	0.857
Triglyceride, mg/dL	183.0 (123.0–295.0)	102.0 (70.0–118.0)	0.005 *

NAS, nonalcoholic fatty liver disease activity score; BMI, body mass index; AST, aspartate aminotransferase; ALT, alanine aminotransferase; GTP, guanosine triphosphate; HDL, high density lipoprotein; LDL, low density lipoprotein. * *P*-value < 0.05.

**Table 2:** Relative expression of tissue and plasma GAS5 according to pathologic results.

Pathologic Feature	Pathologic Grade (Number)		*P*-Value
**Steatosis ≥ 2**	**<2 (21)**	**≥2 (30)**	
Tissue GAS5	4.5 (3.8–6.3)	4.9 (2.6–5.7)	0.602
Plasma GAS5	9.2 (6.6–12.0)	7.9 (5.1–11.2)	0.274
**Inflamation ≥ 2**	**<2 (32)**	**≥2 (29)**	
Tissue GAS5	4.5 (3.3–5.6)	5.3 (3.8–6.8)	0.148
Plasma GAS5	8.1 (6.0–11.3)	9.2 (6.2–11.1)	0.885
**Presence of NASH**	**Not NASH (8)**	**NASH (43)**	
Tissue GAS5	4.2 (2.4–4.6)	5.1 (3.7–5.9)	0.090
Plasma GAS5	8.4 (6.8–10.4)	8.0 (6.0–11.7)	0.990
**Severe NASH**	**Not Severe NASH (27)**	**Severe NASH (24)**	
Tissue GAS5	4.6 (3.7–5.7)	5.2 (3.3–6.3)	0.674
Plasma GAS5	9.2 (6.6–11.3)	7.4 ( 5.0–12.0)	0.448

NASH, nonalcoholic steatohepatitis.

## Supplementary Materials

The following are available online at https://www.mdpi.com/2073-4425/11/5/545/s1, Figure S1. A scheme for the Gas5 primers annealing site.
