# Supplementary figures and images for: Expression of the Long Noncoding RNA GAS5 Correlates with Liver Fibrosis in Patients with Nonalcoholic Fatty Liver Disease
Source: Genes (Basel). 2020 May 13;11(5):545. doi: 10.3390/genes11050545 (PMC7291058; doi:10.3390/genes11050545)

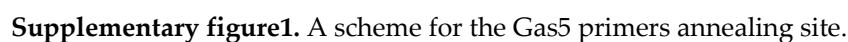

Supplement: Supplementary file 1 [file genes-11-00545-s001.pdf]
